# Mechanism of Hypoxia-Mediated Smooth Muscle Cell Proliferation Leading to Vascular Remodeling

**DOI:** 10.1155/2022/3959845

**Published:** 2022-12-24

**Authors:** Xiaojuan Huang, Elif Ece Akgün, Khalid Mehmood, Hui Zhang, Zhaoxin Tang, Ying Li

**Affiliations:** ^1^College of Veterinary Medicine, South China Agricultural University, Guangzhou 510642, China; ^2^Department of Histology and Embryology, Faculty of Veterinary Medicine, Atatürk University, Erzurum 25030, Turkey; ^3^Department of Pathology, Faculty of Veterinary and Animal Sciences, The Islamia University of Bahawalpur, 63100, Pakistan

## Abstract

Vascular remodeling refers to changes in the size, contraction, distribution, and flow rate of blood vessels and even changes in vascular function. Vascular remodeling can cause cardiovascular and cerebrovascular diseases. It can also lead to other systemic diseases, such as pulmonary hypertension, pulmonary atherosclerosis, chronic obstructive pulmonary disease, stroke, and ascites of broilers. Hypoxia is one of the main causes of vascular remodeling. Prolonged hypoxia or intermittent hypoxia can lead to loss of lung ventilation, causing respiratory depression, irregular respiratory rhythms, and central respiratory failure. Animals that are unable to adapt to the highland environment are also prone to sustained constriction of the small pulmonary arteries, increased resistance to pulmonary circulation, and impaired blood circulation, leading to pulmonary hypertension and right heart failure if they live in a highland environment for long periods of time. However, limited studies have been found on the relationship between hypoxia and vascular remodeling. Therefore, this review will explore the relationship between hypoxia and vascular remodeling from the aspects of endoplasmic reticulum stress, mitochondrial dysfunction, abnormal calcium channel, disordered cellular metabolism, abnormal expression of miRNA, and other factors. This will help to understand the detailed mechanism of hypoxia-mediated smooth muscle cell proliferation and vascular remodeling for the better treatment and management of diseases due to vascular remodeling.

## 1. Introduction

Vascular remodeling has always been a burning issue in cardiovascular disease research, especially in plateau areas. Blood vessels are composed of endothelial cells (ECs), smooth muscle cells (SMCs), and fibroblasts. Vascular remodeling is the dysregulation of migration, proliferation, and apoptosis of these cells, resulting in thickening or thinning of the vessel wall [1]. Hypoxia is very easy to occur in high altitude or low-temperature environment, which leads to vascular permeability increased, intracellular calcium overload, cellular metabolic dysfunction, mitochondrial endoplasmic reticulum (ER) stress, nervous system dysfunction and ultimately leads to vascular remodeling. Hypoxia refers to the pathological process of abnormal changes in tissue and function caused by insufficient intracellular oxygen or oxygen consumption disorder [2]. In a low-temperature environment, mammals are often in a poorly ventilated environment, the concentration of CO_2_ increases and the concentration of O_2_ decreases, and the body needs more oxygen for the normal metabolic function of cells than normal temperature, so mammals are prone to deficiency in low-temperature environments. The hypobaric and hypoxic environment in high-altitude areas can easily lead to the decrease of blood oxygen saturation level and the increase of blood viscosity in mammals and birds, which affects the occurrence of vascular remodeling. Studies have shown that humans living at high altitudes are in a state of chronic hypoxia for a long time, which causes them to have varying degrees of vascular remodeling [3], which seriously affects their health [4]. To date, there are few studies on hypoxia-mediated vascular smooth muscle cell (VSMC) proliferation leading to vascular remodeling. Therefore, this review aims to provide a comprehensive analysis of this to explore some potential prospects.

## 2. Relationship between VSMCs and Vascular Remodeling

Vascular remodeling refers to the process of changes in the size, shape, structure, and function of blood vessels, including apoptosis, proliferation, migration of vascular cells, and the production and degradation of extracellular matrix [5]. VSMCs constitute the middle layer of the vessel wall [6], and its changes can affect vascular remodeling. VSMC can be divided into contractile type and synthetic type according to their different structures and functions [7]. Under normal conditions, VSMC is contractile after maturation, but the differentiation and maturation of VSMC can still be dedifferentiated by some internal environmental factors, such as platelet-derived growth factor BB (PDGF-BB) and angiotensin II (Ang II), and become the synthetic phenotype with low differentiation under stimulation. This process is called SMC phenotypic transformation and is characterized by abnormal cell proliferation, migration, apoptosis, and synthesis [8]. The transformation of VSMCs from contractile type to synthetic type causes excessive proliferation and migration of VSMCs [9, 10], decrease of contractile protein expression [11], thickening of vascular wall, resulting in narrowing of vascular lumen, increased blood pressure, and increased arterial blood vessels. The phenotypic transformation of VSMCs is an important link leading to vascular remodeling, causing hypoperfusion, organ dysfunction, and even end-organ failure [12], which may lead to the occurrence of various diseases, such as pulmonary arterial hypertension (PAH), atherosclerosis, and vascular restenosis. Conventional wisdom holds that VSMC apoptosis is silent, that VSMC undergoes apoptosis as a compensatory mechanism for neoplastic endothelial proliferation and vascular stenosis, and that apoptotic VSMC secrete cytokines or membrane-bound ligands that act via paracrine secretion on adjacent cells to promote cell proliferation and inflammation [13, 14]. The migration of VSMC into the endosomal layer is an important process in the formation of new endosomes [15]. Mature VSMC has the potential to alter their migratory properties and extracellular matrix components, and growth factors, cytokines, and calcium signaling can all promote VSMC migration [16, 17].

## 3. Effects of Hypoxia on VSMCs

Hypoxia can cause a decrease in the partial pressure of oxygen in the blood, which directly affects the contraction of vascular smooth muscle and increases blood pressure. Lower partial pressure of blood oxygen can cause abnormal calcium pumps, abnormal opening of calcium channels, release of vasoactive substances, inflammatory processes, oxidative stress, and matrix deposition triggering a series of biochemical reactions that lead to VSMC survival, proliferation, and migration; thickening of vascular smooth muscle and vascular fibrosis; and promoting the development of vascular remodeling. In addition, hypoxia can prompt the release of inflammatory factors, growth factors, and vasoconstrictor factors from ECs, which induce VSMC proliferation through paracrine secretion. For example, ECs induce VSMC proliferation by secreting large amounts of endothelin-1 (ET-1), Ang II, PDGF-BB, and VEGF-A [18, 19], which induce vasoconstriction and vascular hypertrophy [20]. In addition, VSMC proliferation and migration can be promoted by activating signaling pathways such as mitogen-activated protein kinase signaling (MAPK) [21, 22], protein kinase A (PKA) [23], protein kinase C (PKC) [24], and phosphatidylinositol 3-kinase (PI3K) [25].

## 4. Effects of Mitochondrial Damage and ER Stress on Vascular Remodeling

**Figure 1:**
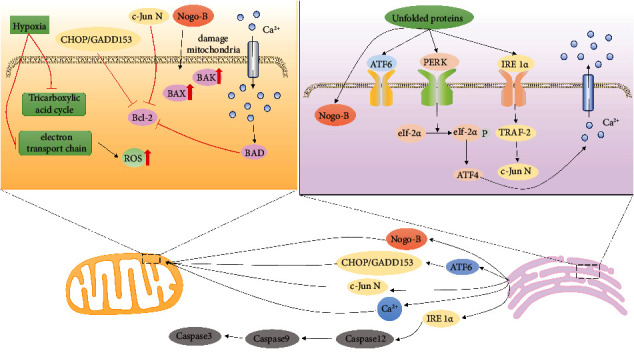
ER stress process induced by hypoxia. ATP production is reduced due to hypoxia, which leads to abnormal protein translation and the release of a large number of [Ca^2+^] from the calcium pool. Finally, the activity of proapoptotic genes is inhibited, and the activity of antiapoptotic genes is activated, which leads to the proliferation of VSMCs.

ER is the largest membrane network structure in cells, and its main function is to synthesize and process proteins. Hypoxia stimulates stress in the endoplasmic reticulum [26, 27]. Hypoxia can lead to inhibition of protein folding and isomerization through activation of IRE 1*α*, PERK, and ATF 6 signaling pathways [28], a process known as unfolded protein response (UFR) (Figure 1), thereby limiting the function of local redox enzyme ERO 1*α* in ER, causing ER stress, increasing the release of inflammatory factors (such as IL-6, tumor necrosis factor-*α* (TNF-*α*), and McP-1), aggravating hypoxia-induced vascular injury, and leading to vascular remodeling [29]. ER has a strong calcium buffer capacity, so it is called the calcium pool. ER calcium concentration is much higher than cytoplasmic calcium concentration under normal conditions. Under the stimulation of hypoxia, the calcium pool releases [Ca^2+^] into the cytoplasm, which increases the concentration of [Ca^2+^] in the cytoplasm and rapidly decreases the concentration of [Ca^2+^] in the ER, which promotes the differentiation cycle of resting cells and makes SMCs proliferate. Under the conditions of ER hypoxia, [Ca^2+^] changes in the internal environment, redox imbalance, and overexpression of nonfoldable defective proteins, the synthetic and posttranslational modified proteins will change, leading to severe accumulation of nonfoldable proteins in ER, resulting in ER stress [30]. ER stress activates the unfolded protein response (UPR), regulates increased Nogo-B expression, and then disrupts ER-like mitochondrial units, resulting in increased mitochondrial membrane potential and mitochondrial superfluidization, promoting [Ca^2+^] inward flow and regulating VSMC migration and proliferation [31], and causing pulmonary artery constriction [32].

Mitochondria are the main sites of glucose oxidation and fatty acid *β*-oxidation in cells. However, hypoxia can directly inhibit aerobic oxidation of mitochondrial glucose oxidation, so the tricarboxylic acid cycle and oxidative phosphorylation are inhibited [33] and alter the production of its products acetyl coenzyme A, reactive oxygen species (ROS), and ATP and induce cell proliferation [26, 27, 34]. Under normoxic conditions, [K^+^] voltage-gated channels remain open, [Ca^2+^] voltage-gated channels are inhibited, and ROS produced by mitochondria are removed by the cellular antioxidant system [35]. In hypoxic conditions, intracellular ROS are elevated, and the antioxidant system is inhibited [36]. In hypoxic conditions, intracellular ROS are elevated, and the antioxidant system is inhibited causing closure of [K^+^] channels, depolarization of the cell membrane, stimulation of [Ca^2+^] channels to open [Ca^2+^] entry into the cell leading to vasoconstriction, and dysregulation of ROS production by mitochondria [37]. Chronic intermittent hypoxia can increase NADPH oxidase-derived ROS by increasing [38]. Complex III produces ROS after hypoxia, and increased oxidation is detected in the mitochondrial intermembrane gap and cytoplasm, while oxidation in the mitochondrial matrix is reduced [35]. ROS inhibit the PHD expression, promote increased hypoxia-inducible factor-1 (HIF-1), and regulate mitochondrial autophagy [39].

Mitochondria not only regulate energy metabolism by controlling the process of oxidative phosphorylation and the levels of mitochondrial enzyme in the cell but also [Ca^2+^] store and regulate cell apoptosis. Insufficient ATP production will inhibit the opening of mitochondrial ATP-dependent [K^+^] channels, resulting in [Ca^2+^] influx, and calcium overload in mitochondria, resulting in mitochondrial damage. Mitochondrial damage triggers the production of ROS, which impacts mitochondria and aggravates the damage of mitochondrial structure and finally leads to the increase of AMP/ATP. ROS induces the transformation of VSMCs into a proliferative phenotype and contributes to vascular hypertrophy and remodeling in hypertension [40].

Mitochondria increase AMP due to hypoxia, and AMP can contribute to the production of ATP through the AMP-activated protein kinase (AMPK) pathway in glycolysis [21, 22]. The AMPK pathway can promote the glycolysis process to produce ATP. Abnormal energy metabolism can also affect ER homeostasis and produce abnormal synthesis and processing proteins of ER [41].

## 5. Effect of Hypoxia on [Ca^2+^] Channel

The contraction of vascular smooth muscle is caused by the increased free calcium concentration in cells promoting the formation of actin myosin cross bridge [42]. Hypoxia affects mitochondrial ROS production and causes altered redox-sensitive [K^+^] and [Ca^2+^] channel activity in the cell membrane [43]. [Ca^2+^] can enter VSMC from outside the cell via voltage-gated channels and nonvoltage-gated channels [30]. The phenotypic switch of VSMCs is also related to the calcification process [5]. [Ca^2+^] homeostasis is an important factor regulating cell division, proliferation, and vascular activity. Intracellular calcium overload can cause abnormal cell excitation-contraction coupling mechanism, resulting in abnormal vascular function.

Hypoxia directly inhibits voltage-dependent [K^+^] channels, decreases [K^+^] efflux, decreases cell membrane potential, and causes abnormal opening of L- and T-type voltage-dependent [Ca^2+^] channels in VSMC (Wu, et al., 2021). Hypoxia can cause abnormal opening of [Ca^2+^] channels in VSMCs [44, 45] and [Ca^2+^] influx, with the increase of calcium concentration, and calpain is activated to promote the proliferation of VSMCs and vascular remodeling by upregulation of calpain-1, -2, and -4 [46].

Store-operated calcium channels (SOCCs) are the main channels for [Ca^2+^] to enter PASMC under anoxic conditions [47]. SOCC is located on the SMC membrane. When [Ca^2+^] from ER and stromal reticulum are released into the cytoplasm, the calcium channels on the cell membrane and the ON extracellular [Ca^2+^] are also opened. SOCC consists mainly of transient receptor potential (TRP), Orai protein and stromal interaction molecule (STIM), and caveolin-1 [48]. Hypoxia causes upregulation of transient receptor potential vanilloid 3 (TRPV3), which induces an increase in proliferating cell nuclear antigen (PCNA) and regulates the cell cycle by regulating cell cycle proteins and phosphorylated CaMK II (p-CaMK II) and activating the phosphatidylinositol-3-kinase (PI3K)/Akt pathway to increase intracellular calcium concentration [49]. In addition, hypoxia promotes upregulation of intracellular [Ca^2+^] concentrations through activation of transient receptor potential canonical (TRPC) channels [50, 51]. For example, hypoxia upregulates TRPC6, which causes acute pulmonary vasoconstriction through receptor action on calcium channels and pulmonary vascular remodeling through storage action on calcium channels [50, 51]. STIM 1 is a single channel protein located on the ER and sarcoplasmic reticulum, which can be used as a calcium sensor of the ER and sarcoplasmic reticulum [52]. In the absence of oxygen and in higher reservoir of [Ca^2+^], phospholipase C (PLC) hydrolyzes phosphatidylinositol diphosphate (inositol-1,4,5-triphosphate, IP3) and diacylglycerol (DAG) [53]. IP3 binds to the IP3 receptor on the ER. The ER releases large amounts of [Ca^2+^] into the cytoplasm, leading to depletion of calcium stores. When the concentration of [Ca^2+^] in the ER and sarcoplasmic reticulum decreases, STIM 1 transfers from the ER and sarcoplasmic reticulum to the membrane and binds to the Orai protein, resulting in calcium influx and smooth muscle contraction [54].

## 6. Effect of Cytokines on VSMCs

Vascular smooth muscle contraction is regulated not only by calcium-dependent mechanisms but also by noncalcium-dependent mechanisms, including a-Ras-homologous kinase (Rho A), protein kinase C (PKC), and mitogen-activated protein kinase (MAPK) [55]. Hypoxia can promote mast cells, macrophages, and vascular ECs to release a variety of cytokines and chemokines [56], some of which can contract pulmonary blood vessels, such as leukotriene, thromboxane A_2_, prostaglandin, endothelin-1 (ET-1), Ang II, and IL-1 and -6. These vasoconstriction substances can lead to vasoconstriction, VSMC proliferates uncontrollably, and elevated blood pressure and a series of vascular wall lesions can exacerbate vascular remodeling. Leukotrienes are mainly synthesized by the Golgi apparatus and ER of leukocytes, and excessive release of LTB4 from activated macrophages can cause PASMC proliferation [57]. Broad pathological effects of leukotrienes include increased production of matrix proteins, increased smooth muscle contractility and proliferation, and enhanced cell survival [58]. Thromboxane A_2_ binds to the receptor TP and causes [Ca^2+^] inward flow via the PLC/IP3 pathway [Ca^2+^] contraction in smooth muscle [59, 60]. Prostaglandin D2 (PGD 2) receptor subtype 1 (DP 1) is a receptor for PGD 2. Under hypoxia, PGD 2 is reduced, resulting in DP 1 loss, which intensifies hypoxia-induced vascular remodeling. DP1 deficiency promotes VSMC hypertrophy and proliferation by enhancing mammalian target of rapamycin complex (mTORC) activity. DP1 activation promotes dissociation of the mTORC1 complex and inhibits mTORC1 activity via phosphorylation of PKA at Ser791 [61, 62]. Ang II and hypoxia both induce VSMCs to produce ET-1, which activates G protein-coupled receptors on VSMC membranes and causes VSMCs to produce ROS which causes vasoconstriction. Actions of Stamp 2 in macrophages drive vascular remodeling processes in SMCs via secreted factors such as CXCL 12 [63].

**Figure 2:**
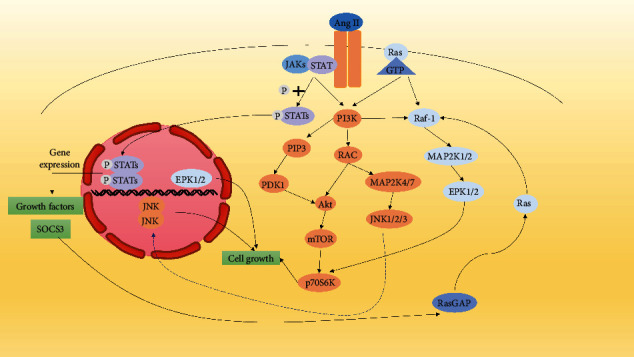
Ang II promotes SMC proliferation mainly through JAK, PI3K/Akt, and MAPK pathways. (1) Ang II promotes cells growth, proliferation, and survival through the Raf-1/MAPK pathway. (2) Ang II activates receptor JAKs and stimulates phosphorylation of STATs, which translocate to the nucleus and bind to SIE elements to promote transcription and translation of cell proliferation genes [69]. (3) Ang II stimulates cells proliferation via the PI3K/AKT/mTOR pathway.

Ang II is one of the cytokines that play an important role in vascular remodeling, and the effect of Ang II on VSMCs is exemplified by the fact that Ang II is the most important endocrine ligand in the renin-angiotensin system (Ras) [64, 65]. Ang II can stimulate VSMC proliferation through the MAPK signaling pathway. The signaling pathway is as follows: Ang II binds to the receptor AT_1_R and activates the PI3K/PK-MEK-ERK1/2 pathway [16, 17], and Ang II acts directly on VSMCs, causing VSMC proliferation [66]. Activation of AT_1_R activates the receptor tyrosine kinase (JAK), which promotes VSMC proliferation via the JAK/STAT pathway. Ang II stimulates the epidermal growth factor receptor (EGFR) to activate the Ras/ERK cascade and PI3K/Akt/p70S6K kinase cascade and the ER stress/unfolded protein response that causes VSMC proliferation [67] (Figure 2). In addition, AT_1_R is also a heterodimeric G protein-coupled receptor (GPCR), which causes initial heterodimeric G protein dissociation and activation of ligand-specific intermediates (including nonreceptor tyrosine kinases) by activating EGFR, resulting in increased intracellular [Ca^2+^] concentrations and promoting mitochondrial production of ROS [68].

## 7. Effect of MicroRNAs on VSMCs

Hypoxia induces the expression of specific microRNAs (miRs) in VSMCs. miRs are small noncoding RNAs, approximately 21-23 nucleotides in length, that repress the expression of targeted genes by binding to the 3′ untranslated region of the target gene [70]. In addition, many studies have found that miRNA is related to VSMC proliferation and vascular remodeling [71]. Here are some examples of which miRNA are involved in vascular remodeling.

Under hypoxic conditions, miR-1 is downregulated in VSMCs and promotes sphingosine kinase 1 expression to inhibit apoptosis [72]. The low expression of miR-1 underlies hypoxia-induced VSMC proliferation and migration. miR-17 is upregulated in VSMCs under hypoxia, and miR-17 regulates VSMC proliferation and apoptosis through Mfn 2 [73]. miR-18a-5p is upregulated in pulmonary artery smooth muscle of PAH patients. miR-18a-5p promotes VSMC proliferation and migration by repressing the target gene Notch2 [74]. Hypoxia induced upregulation of miR-19a in pulmonary arteries and inhibited phosphatase and tensin homolog deleted on chromosome ten (PTEN) expression to promote VSMC proliferation and migration; in addition, HIF-1*α* also promoted miR-19a upregulation [75]. miR-150 is downregulated in the pulmonary arteries of PAH patients. miR-150 inhibits the expression of *α*-SMA, a marker of PASMC, and alters the overproliferation of growth factor-*β* (TGF-*β*), mainly through the Akt/mTOR signaling pathway [76]. Hypoxia-induced elevation of miR-155-5p levels in PASMCs affects cell cycle progression by directly targeting glycogen phosphorylase to regulate cell cycle protein D1, cell cycle protein E, and CDK2 [77]. miR-760 is reduced in the pulmonary arteries of PAH patients, and miR-760 regulates hypoxia-induced VSMC proliferation, migration, and apoptosis by targeting toll-like receptor 4 [78].

In VSMCs of hypoxia-induced PAH tissue, the expression of miRNA-140-5p and SOD 2 was inhibited, while the expression of Dnmt 1 was increased, which promoted VSMC proliferation and inhibited apoptosis and differentiation [79]. Under hypoxic conditions, miRNA-143 and miRNA-145 were upregulated, ATP-binding cassette transporter A1 (ABCA 1) was downregulated [79], and miRNA-143/145 could promote hypoxia-induced VSMC proliferation and migration by inhibiting the ABCA 1 overexpression [80]. miRNA-145 can also inhibit VSMC proliferation, migration, and phenotypic switch by preventing the activation of PI3K/Akt/mTOR signaling pathway [81]. miRNA-145 can induce VSMC phenotypic differentiation by promoting TGF-*β*.

Under hypoxic conditions, miRNA-26b-5p was found to be significantly downregulated, and TGF was regulated through the Smad4-*β*/Smad 4 signaling pathway, promoting phenotypic transformation and TGF-*β* expression in VSMCs. Upregulation of TGF-*β* has a regulatory function on differentiation and proliferation of SMCs. [82]. Hypoxia induces upregulation of miRNA-92b-3 and miRNA-214, miRNA-92b-3 promotes VSMC proliferation through mTOR signaling pathway [83], and miR-214 promotes cell proliferation by inhibiting the expression of CCNL 2 [84]. Studies have shown that hypoxia upregulates the expression of cullin 7 mRNA and miR-1260b, cullin 7 mRNA leads to the proliferation and migration of VSMCs by downregulating the expression of p53 [50], miR-1260b promotes the proliferation and migration of VSMCs by downregulating the expression of growth differentiation factor 11 (GDF 11), and 3′ UTR binds to inhibit the expression of GDF 11, thereby inhibiting Smad signaling and enhancing VSMC proliferation [85] and promoting vascular remodeling. miR-223 was significantly downregulated under hypoxia, and the reduction of miR-223 promoted Rho B to regulate the proliferation, migration, and expression of myosin light chain in VSMCs, thereby affecting vascular remodeling and PAH development [86].

## 8. Transcription Factors Are Involved in Hypoxia-Induced Vascular Remodeling

Transcription factors can bind to specific sequences upstream of the 5′ end of genes, and they are protein molecules that mediate cell proliferation, differentiation, apoptosis, inflammation, and immune signal transduction. Under hypoxic conditions, abnormal increase or decrease of cytokines, growth regulators and vasoactive substances will affect the expression of transcription factors, regulate other cellular regulators, and thus participate in the proliferation of VSMCs. Transcription factors such as macrophage migration inhibitory factor, HIF-1, and Ras can bind to the regulatory sites of promoters to promote the production of VSMC proliferation-related growth factors.

### 8.1. Effect of HIF-1 on VSMCs

Hypoxia can mediate [Ca^2+^] channel opening, mitochondrial fission, increased ET-1 secretion, and vascular endothelial-derived growth factor expression through upregulation of the HIF-1 expression, leading to the development of vascular remodeling [87]. HIF-1 is a transcriptional regulator. HIF-1 is a heterodimer composed of HIF-1*α* and HIF-1*β* subunits, which can regulate the expression of multiple genes and help maintain the stability of the intracellular environment under hypoxic conditions. miRs are small noncoding RNAs of HIF-1 and are readily hydrolyzed and inactivated by the prolyl hydroxylase domain (PHD) at normal oxygen concentrations in cells [88]. PHD is a member of the 2-ketoglutarate- (2-OG-) dependent dioxygenase superfamily, whose hydroxylation activity is catalyzed by oxygen, iron ions, and 2-OG. PHD as a key enzyme regulating HIF can be hydroxylated and lead to its degradation, thus affecting the outcome of related diseases [89]. Under normoxic conditions, PHDs can hydroxylate the prolyl group of HIF-1*α*, and then von Hippel-Lindau protein (VHL) promotes the degradation of HIF-1*α* [90].

However, PHD is inhibited under hypoxic conditions and fails to hydroxylate the HlF-1 subunit, which promotes the formation of HIF transcription factors [91]. Its half-life is prolonged under hypoxia environment, thus further activating downstream signaling pathways and playing a biological role [92]. HIF-1 controls oxygen transport by promoting angiogenesis and glycolysis processes. HIF-1 inhibits oxygen consumption and promotes glycolysis through pyruvate dehydrogenase kinase 1 (PDK1), promoting the expression of ROS, while HIF-1 activation enhances cell resistance to death [93]. ROS are highly active molecules in the body, including superoxide anion, hydroxyl radical, and hydrogen peroxide. ROS produced by hypoxia can cause continuous contraction of SMCs and upregulate the expression of various proliferation-related genes together with HIF-1 to promote SMC proliferation and migration [94].

**Figure 3:**
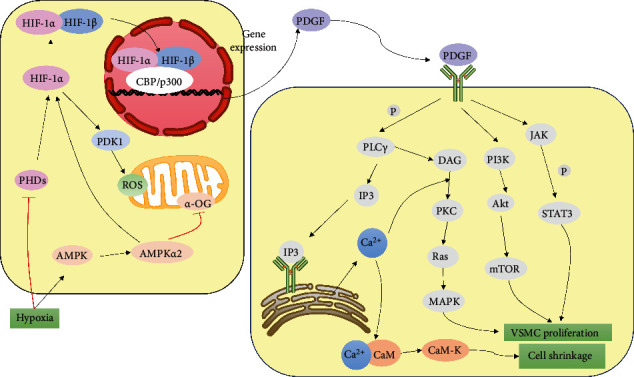
Hypoxia can lead to the effect of AMPK pathway, and the increased expression of HIF-1 promotes the effect on SMCs. (1) Hypoxia inhibits mitochondrial production of *α*-OG by promoting the AMPK pathway, which inhibits the expression of PHDs and promotes the expression of HIF-1*α*. (2) Hypoxia inhibits the expression of PHDs, resulting in stable expression of HIF-1*α* and increased expression of PDK1, which promotes the expression of ROS, which in turn inhibits the hydroxylation of HIF-1*α* by PHDs. (3) HIF-1*α* and HIF-1*β* translocate to the nucleus to bind to hypoxic elements and regulate the transcription and translation of genes related to cell proliferation [104]. (4) HIF-1 promotes PDGF expression and PDGF binds to corresponding receptors to activate SOCC, MAPK, PI3K/Akt, and JAK/STAT3 signaling pathways [105]. (5) PDGF induces transactivation of cyclin D1 and survival proteins in SMC through phosphorylation of STAT3, thereby promoting SMC proliferation and migration and reducing apoptotic cell death [106]. (6) PDGF induces the breakdown of phosphatidylinositol 4,5-bisphosphate (PLC*γ*) to IP3 and diacylglycerol (DAG), which binds to receptors on the ER and stimulates the release of [Ca^2+^] from the calcium pool to the cytoplasm [103]. DAG stimulates VSMC proliferation via the PKC/Ras/MAPK pathway.

HIF-1*α* promotes vascular remodeling by participating in the production of vascular endothelial growth factor (VEGF). The relative expression level of VEGF mRNA in SMCs increased significantly within a few hours after hypoxia and then decreased to the normal level after the resumption of oxygen supply, indicating that hypoxia can lead to the increase of VEGF gene expression in cells, resulting in the proliferation and migration of SMCs [95]. VEGF binding to VEGFR can cause extracellular [Ca^2+^] influx and VSMC migration [96, 97]. VEGF promotes smooth muscle phenotype transformation through activation of STAT3 leading to SMC cell proliferation [98]. VEGF also causes increased intracellular ROS, NF-*κ*B activation, and IL-6 expression [99]. HIF-1 also promotes the expression of PDGF [100]. PDGF induces VSMC proliferation through the MAPK and PI3K/Akt/protein kinase B (PKB) signaling pathway, which controls cell cycle progression and protein and DNA synthesis [101]. In addition, PDGF activates the signal transducer and activator of transcription (STAT) protein family and binds specifically to the regulatory elements SIE or GAS [102]. PDGF activates SOCC by phosphorylating PLC-*γ* [Ca^2+^] depletion in the calcium pool that causes STIM to translocate to the cell membrane and bind to Orai1 protein, opening [Ca^2+^] channels and [Ca^2+^] inward flow [103] (Figure 3).

### 8.2. Effect of Rho Kinases on VSMCs

Rho kinases belong to the serine/threonine kinase family, and the serine/threonine kinase Rho kinase (ROCK) mediates a variety of cellular responses related to PAH [107]. Rho kinase causes excessive vascular smooth muscle contraction and vascular remodeling through inhibition of myosin phosphatase and activation of its downstream effectors [108]. Wang et al. reported that PASMC exposed to hypoxia showed increased Rho kinase activity and increased phosphorylation of myosin light chains, causing sustained contraction of vascular smooth muscle [99, 109]. The Rho/ROCK pathway plays an important role in a variety of essential cellular functions, including contraction, motility, proliferation, and migration, leading to the development of cardiovascular disease. Rho A and Rho C activate the ROCK signaling pathway by phosphorylating the protein MYPT-1, thereby regulating the proliferation and migration of VSMCs [110]. ROCK2 regulates VSMC contractility through direct binding and phosphorylation of myosin phosphatase [111]. During hypoxia, activation of ROCK enhances NHE activity and promotes migration and proliferation of PASMC. Activation of Rho kinase (ROCK) by endothelin-1 enhances NHE activity in PASMC and leads to pH alkalinization [112]. Ang II also regulates the Rho A/ROCK signaling pathway and actin polymerization via the AT_1_R, which then affected the dedifferentiation of VSMCs [113].

### 8.3. Effect of TRAIL, NHE, and HIMF on VSMCs

TNF-related apoptosis-inducing ligand (TRAIL) is a type II transmembrane protein, and TRAIL mRNA and protein are mainly expressed in SMCs of the pulmonary artery and aorta [114]. TRAIL binds to the receptor TRAIL-R and causes VSMC proliferation and survival by activating the Akt, NF-*κ*B, and EPK pathways [115].

Intracellular pH homeostasis is key to VSMC function. The Na^+^/H^+^ exchanger (NHE) is an important mechanism in the regulation of cellular pH. Hypoxia causes an increase in VSMC pH [116]. NHE is an integral membrane protein that transports intracellular [H^+^] out of cells and extracellular [Na^+^] into cells, thereby protecting cells from intracellular acidification [117]. VSMCs exposed to hypoxia in vivo or in vitro exhibited elevated pH and enhanced NHE activity [118]. The increase of pH can affect the increase of vascular pressure and can also promote the increase of intracellular [Ca^2+^] concentration by regulating the activity of calmodulin-dependent enzymes [116]. During hypoxia, activation of HIF-1 and ROCK enhances NHE activity and promotes VSMC migration and proliferation [44, 112, 119, 120].

**Figure 4:**
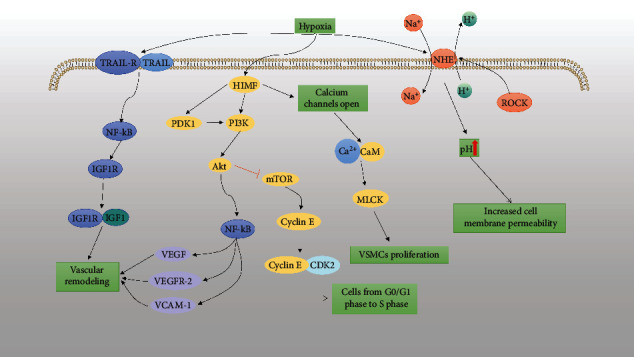
Effect of TRAIL, NHE, and HIMF on VSMCs. (1) Hypoxia induces TRAIL to combine with TRAIL-R and enhance the expression of insulin-like growth factor-type 1 receptor (IGF1R) through the NF-*κ*B pathway, promoting vascular intimal hyperplasia [125]. (2) HIMF upregulates VEGF and VCAM expressions via PDK1/PI3K/Akt/NF-*κ*B signaling pathway [126]. (3) HIMF regulates the cell cyclin E expression through the PI3K/AKT/mTOR pathway, causing a rapid shift from G0/G1 phase to S phase development in VSMCs [127]. (4) Hypoxia-induced increase in the HIMF expression results in the release of large amounts of [Ca^2+^] from the calcium pool into the cytoplasm [94, 128]. (5) Hypoxia activates the NHE channel, resulting in a decrease in intracellular [H^+^] and an increase in [Na^+^], leading to a rise in pH and VSMC proliferation.

Hypoxia-induced mitogenic factor (HIMF) is also known as inflammatory zone 1 or resistin-like molecule-*α* [121]. The HIMF expression was low in normoxic state but significantly increased in vascular ECs, SMCs, bronchial epithelial cells, and type II alveolar epithelial cells under hypoxia state. HIMF strongly activated Akt phosphorylation. It promotes SMC proliferation through the PI3K/Akt pathway (Figure 4) [122]. HIMF increases vascular pressure and vascular resistance more potently than either ET-1 or Ang II. Hypoxia upregulates [Ca^2+^]-sensitive receptor and HIMF expression through activation of the Rho A/ROCK2 pathway [123]. HIMF can stimulate [Ca^2+^] release from the calcium pool in VSMC via the PLC/IP3 signaling pathway [124].

### 8.4. Effect of Cyclin on VSMCs

Cyclin is a protein whose concentration varies with the cell cycle of eukaryotic cells and includes cyclin A, cyclin B, cyclin D, cyclin E, cyclin G, and cyclin H. They are involved in regulating the enzymatic activity of cyclin-dependent kinases (CDKs) to regulate cell cycle processes. Hypoxia also promotes VSMC viability; increases the expression of PCNA, cyclin D, cyclin E, cyclin A, and p-CaMK II; moves VSMCs from G0/G1 phase to G2/M + S phase; enhances microtubule formation; and increases the number of intracellular [Ca^2+^] [9]. Cyclin D1 reduces the time to S phase, accelerates the G1/S transition, and promotes cell proliferation [77]. Cyclin D1 can be expressed through EPK signal pathway. Macrophage migration inhibitory factor upregulates cyclin D1 via ERK signaling, induces VSMC proliferation, and leads to vascular remodeling [129]. Hypoxia promotes the expression of proliferating cell nuclear antigen (PCNA), cyclin E, and cyclin A; changes VSMCs from G0/G1 phase to S phase; and also inhibits the expression of Storkhead box (Stox 1) in VSMCs by activating Akt protein [130]. PCNA exists in the nucleus and is a helper protein of DNA polymerase and plays an important role in initiating cell proliferation. Under hypoxic conditions, the increased expression of the mitophagy protein PINK 1/Parkin induces VSMC proliferation, inhibits apoptosis, and ultimately leads to vascular remodeling, leading to pulmonary hypertension [131]. Deletion of nucleotide-binding oligomerization domain protein 2 (NOD 2) exacerbates hypoxia-induced proliferation, vascular remodeling in PAH and VSMC, and promotes HIF-1*α* expression and Akt phosphorylation [132].

*β*-Catenin is widely found in various types of cells. Its main functions are mediating intercellular adhesion and participating in gene expression as well as in regulating cell proliferation, differentiation, and apoptosis. *β*-Catenin is a bifunctional protein that plays an important role in regulating the transcription of c-Myc and cyclin D1. Aquaporin 1 (AQP 1), a protein located in the red cell membrane, forms “channels” in the cell membrane that control the flow of water in and out of the cell, acting as the “pump of the cell.” It can affect contraction and expansion of red blood cells. The AQP1 overexpression upregulates *β*-catenin protein levels, enhances the expression of *β*-catenin targets, and promotes VSMC proliferation and migration [133]. SphK 1 plays an important role in cell growth, differentiation, and programmed death by regulating the metabolic homeostasis of sphingosine. Sphk 1 catalyzes the production of 1-sphingosine, which promotes cell proliferation and angiogenesis [134].

## 9. Molecular Mechanisms of Other Potential Factors Involved in Hypoxia-Induced Vascular Remodeling

NO is mainly produced by ECs, and hypoxia stimulates the production of NO by EC, which diffuses to VSMC and then acts as an important regulator of VSMC proliferation by inducing the production of cleaved cysteine aspartame and p21 expression [135, 136]. TGF-*β* promotes SMC proliferation through upregulation of the Nox4 expression, which is the main isoform expressed in PASMCs of patients with pulmonary hypertension, and Nox4 may affect VSMC proliferation by promoting ROS production [137]. In addition, hypoxia stimulates sympathetic nerve release of serotonin and epinephrine, and serotonin increases [Ca^2+^] in SMCs [138], increases epinephrine release, and causes proliferation of SMCs through the renin-angiotensin axis, leading to vascular remodeling [139]. Hypoxia causes enhanced glycolysis, leading to increased lactate production. Lactate causes the VSMC phenotype to switch to the synthetic phenotype by enhancing the activity of the lactate transporter protein [140].

## 10. Conclusion and Future Outlook

Studies have shown that elevated calcium ion concentration, synthesis, and secretion of angioconstrictor such as growth factors Ang II and ET and inhibition of apoptosis-related mRNA expression can contribute to the development of vascular remodeling. MAPK signaling pathway and sympathetic nerve excitation can effectively inhibit the proliferation, and sympathetic nerve excitation can effectively inhibit the proliferation of SMCs and vascular remodeling caused by hypoxia. However, many experiments have found that hypoxia can induce the expression of many cytokines and miRNAs. Various types of miRNAs are involved in regulating SMC proliferation and vascular remodeling. The role of HIMF is involved in the pathogenesis of diseases that has not been completely explored, and whether there are other ways to promote SMC proliferation and vascular remodeling, etc., all these mechanisms are needed to be further studied. Today, remodeling and inappropriate vasoconstriction diseases are often treated for their symptoms, and there is no effective primary treatment for the cause [141]. Therefore, more studies are needed to deepen the understanding of the process of SMC proliferation and vascular remodeling caused by hypoxia and provide better ideas for the treatment of pulmonary artery remodeling, pulmonary hypertension, and other diseases caused by hypoxia.
